# Quantitative myocardial perfusion performs as well as visual analysis in diagnosing myocardial ischaemia: a CE-MARC sub-study

**DOI:** 10.1186/1532-429X-17-S1-P93

**Published:** 2015-02-03

**Authors:** John D Biglands, Montasir Ibraheem, Derek R Magee, Aleksandra Radjenovic, Sven Plein, John P Greenwood

**Affiliations:** Division of Medcial Physics, Leeds Institute of Cardiovascular and Metabolic Medicine, University of Leeds, Leeds, UK; Department of Medical Physics and Engineering, Leeds Teaching Hospitals NHS Trust, Leeds, UK; School of Computing, University of Leeds, Leeds, UK; Institute of Cardiovascular and Medical Sciences, College of Medical, Veterinary and Life Sciences, University of Glasgow, Glasgow, UK; Multidisciplinary Cardiovascular Research Centre & Leeds Institute for Cardiovascular and Metabolic Medicine, University of Leeds, Leeds, UK

## Background

Visual analysis of first-pass perfusion CMR studies for assessing myocardial perfusion has been shown to have high diagnostic accuracy for coronary artery disease (Greenwood et al., Lancet, 2012). The aim of this study was to compare the diagnostic performance of quantitative myocardial blood flow (qMBF) estimates with visual analysis on a representative subset of the CE-MARC study.

## Methods

This was a retrospective study using a 128 patient sub-sample of patients, selected from the CE-MARC study such that the distribution of risk factors and disease status within the sample was representative of the full study population. Both visual and quantitative analysis were carried out using the AHA standardised 16 segment model. Visual analysis was part of the original CE-MARC study and was performed by 2 expert reviewers in consensus. To obtain qMBF estimates, manual contouring of the myocardium was carried out and individual qMBF estimates were obtained for each AHA segment using Fermi-constrained deconvolution. The arterial input function was taken from the left ventricular blood pool of the basal slice. Myocardial perfusion reserve values were calculated on a segment by segment basis by dividing the stress by the rest qMBF. The reference standard for myocardial ischaemia was a quantitative coronary X-ray angiogram (QCA) score of ≥70% in any of the coronary territories, or ≥50% in the left main stem. Diagnostic performance was calculated using receiver operator characteristic (ROC) curve analysis. For visual analysis the summed score from all AHA segments was used as the diagnostic measure. For quantitative analysis the minimum MPR value from the AHA segments was used. A DeLong, DeLong, Clarke-Pearson analysis was performed to test for a statistically significant difference in the area under the curve (AUC) values between visual and quantitative analysis.

## Results

The AUC for visual analysis was 0.88 CI: 0.81 to 0.95 with a sensitivity of 81.0% CI: 69.1% - 92.8% and specificity of 86% CI: 78.7% - 93.4%. For quantitative analysis using MPR the AUC was 0.86 CI: 0.79 to 0.93 with a sensitivity of 87.5 CI: 77.3%-97.7% and specificity of 83.3% CI: 75.4%-91.3%.

## Conclusions

This study did not find a statistically significant difference in diagnostic performance between visual and quantitative analysis. This suggests that quantitative analysis performs as well as expert visual analysis and could be a useful measurement for consideration alongside, or even as a replacement for, visual analysis clinically, with the potential of being less observer and expertise dependent.

## Funding

During this work S Plein was funded by a British Heart Foundation fellowship (FS/10/62/28409). JP Greenwood and S Plein received an educational research grant from Philips Healthcare. A Radjenovic and DR Magee were partially supported by WELMEC, a Centre of Excellence in Medical Engineering funded by the Wellcome Trust and EPSRC, under grant number WT 088908/Z/09/Z. JD Biglands was funded by and NIHR research training fellowship (NIHR/RTF/01/08/014).

**Figure 1:**
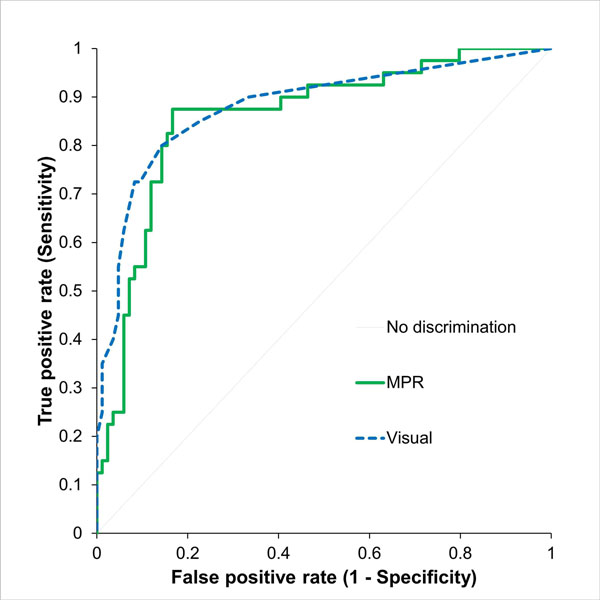
ROC curves for visual and quantitative analysis. No significant difference in diagnostic performance was observed (p=0.63)

